# Pregnancy Outcomes and Maternal Periodontal Diseases: The Unexplored Connection

**DOI:** 10.7759/cureus.61697

**Published:** 2024-06-04

**Authors:** Sarah Mariam, Shamimul Hasan, Mrunal Shinde, Juhi Gupta, Sajad A Buch, Komal S Rajpurohit, Vishakha Patil

**Affiliations:** 1 Department of Periodontology, Bharati Vidyapeeth (Deemed to be University), Pune, IND; 2 Department of Oral Medicine and Radiology, Faculty of Dentistry, Jamia Millia Islamia, New Delhi, IND; 3 Department of Conservative Dentistry and Endodontics, Bharati Vidyapeeth (Deemed to be University), Pune, IND; 4 Department of Oral Medicine and Radiology, Dr. Ziauddin Ahmad Dental College, Aligarh Muslim University, Aligarh, IND; 5 Department of Clinical Oral Health Sciences, School of Dentistry, IMU University, Kuala Lumpur, MYS

**Keywords:** public health, pregnancy, periodontitis, maternal, adverse birth outcomes

## Abstract

In the early 20th century, numerous in-vitro studies, animal studies, epidemiological studies, and human trials have attempted to demonstrate the interrelationship between pregnancy outcomes and maternal periodontal disease. This review aims to shed light on the unexplored connections between pregnancy outcomes and maternal periodontal diseases. A literature search was conducted using electronic databases such as PubMed, Scopus, Google Scholar, Web of Science, and Embase. Our research focuses on the role of epigenetics, maternal vitamin D status, stress levels, genetic factors, innate immunity, pattern recognition receptors, and any potential paternal influence, and their possible connections to maternal periodontal disease. Although the precise etiologies and pathogenic mechanisms of the adverse pregnancy outcomes remain obscure, substantial affirmation of the inter-relationship between maternal periodontal diseases and adverse pregnancy outcomes may prove to be of public health relevance as periodontitis can certainly be prevented and treated. Maternal periodontal disease may augment the probability of jeopardizing maternal health causing adverse effects on the pregnancy and neonatal morbidity. Hence, emphasis should be placed on an early diagnosis and management of periodontal diseases. Routine oral health evaluation during prenatal care should be encouraged to combat complications. Ensuing endeavors should be undertaken to help find plausible mechanisms keeping in view the future research domains and new pathways.

## Introduction and background

Periodontal medicine is an emerging branch of periodontology that focuses on the effect of periodontal inflammation on systemic health. It has opened new avenues for the exploration of oral-systemic interlink. The scientific basis for periodontal medicine comes from the triad of epidemiology, systems biology, and epigenetics. Offenbacher et al. first identified a link between maternal periodontal disease and pregnancy outcomes. These outcomes include preterm labor, low-birth weight, gestational diabetes, preeclampsia, intrauterine growth retardation, and fetal death [1].

Periodontal diseases comprise a large group of disorders of the periodontal tissues of a predominant infectious/inflammatory origin. Gingivitis, the most common form of gingival inflammation, is a reversible inflammatory reaction of the dentogingival tissues to bacterial plaque accumulation, which resolves soon after the dental bacterial biofilm is disrupted. Periodontitis, in contrast to gingivitis, is a chronic inflammatory reaction of the same compartment involving not only superficial gingival tissues but also the periodontal ligament and alveolar bone [2]. The prevalence of gingivitis varies between 50% to 90% among all adults worldwide [3]. The prevalence of periodontitis is reported to be between 20% and 50% of the worldwide population [2].

The American Academy of Periodontology defines periodontitis to be typified by bleeding on probing, gingival recession, pocket formation, and alveolar bone loss [4-6]. Periodontitis is caused by periodontal pathogens mainly gram-negative bacteria, and *Porphyromonas gingivalis* is a “keystone pathogen” for periodontal diseases. Moreover, it is also an immune modulatory disease where various inflammatory mediators play a role in its causation [1]. Numerous epidemiological studies have provided evidence in favor of two mechanisms: (a) the role of oral microbes traveling to the fetal placental unit inducing inflammatory reactions and (b) the role of inflammatory cytokines mainly prostaglandins, which induce preterm labor [7].

*P. gingivalis* and *Fusobacterium nucleatum* are the most common species of periodontal pathogens found in the fetal-placenta unit. The unique virulence factors, surface adhesions, and enzymes of *P. gingivalis* elicit direct or indirect damage to fetal and maternal tissue, resulting in dysfunction of the maternal endothelium and eventually leading to the occurrence of systemic inflammatory responses. *P. gingivalis* can manipulate the balance between different immune cells, enhancing its persistence and survival in maternal and fetal tissues. This capability also improves its ability to evade immune responses. *P. gingivalis* can lead to increased production of pro-inflammatory cytokines and activation of acute-phase responses, which causes a shift in the maternal-fetal immune response and potentially results in adverse pregnancy outcomes [8].

There is a substantial global burden of periodontal diseases, and they may have accompanying detrimental influences on pregnancy outcomes; hence, it is imperative to elucidate their association. Thus, efforts should be made to prevent periodontal diseases by encouraging maintaining good oral hygiene during pregnancy. Treatment strategies should also be instituted to manage periodontal diseases during pregnancy, thereby diminishing the incidence of unfavorable pregnancy outcomes [9].

Studies have investigated the occurrence of periodontal disease during pregnancy, yielding a wide variation in prevalence (11%-100%) [10,11]. Published literature has also explored probable connotations between maternal periodontal diseases and adverse pregnancy consequences but with conflicting results. These conflicting results may have arisen because of wide variation among the sample subjects, there is a high degree of variability in study populations, as well as in recruitment and evaluation procedures. Another possible reason for these inconsistent results may be the potential unfavorable exposures shared commonly between periodontitis and adverse pregnancy consequences [12].

**Figure 1:**
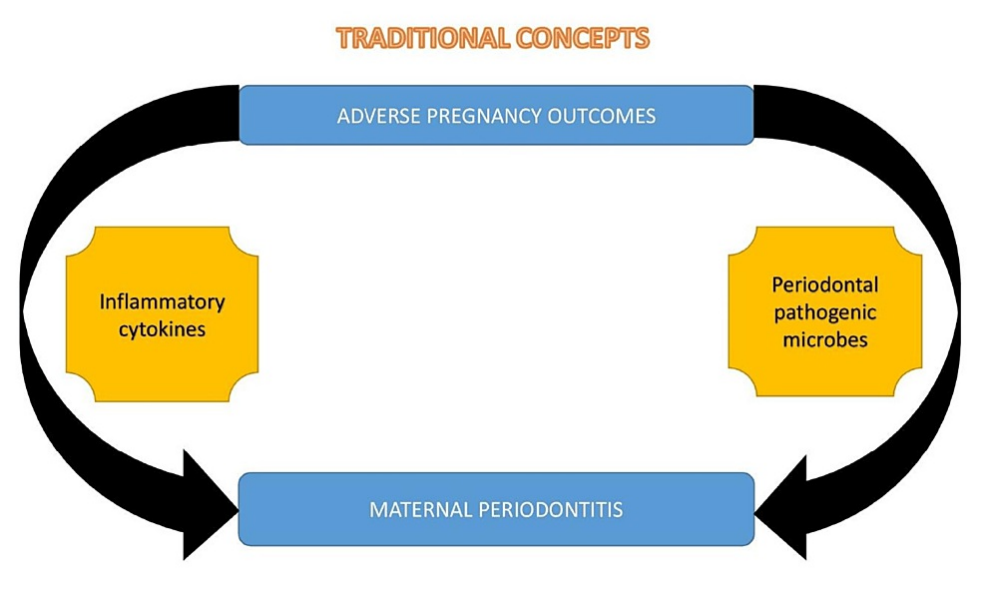
Traditional concept of maternal periodontitis and adverse pregnancy outcomes. Image credit: Dr. Sarah Mariam

The traditional concept of maternal periodontitis on adverse pregnancy outcomes is illustrated in Figure 1.

## Review

Recent research has focused on the epigenetic links, stress levels, maternal vitamin D status, genetic factors, role of innate immunity, role of pattern recognition receptors (PRR), paternal influence (if any), and their linkage with maternal periodontal disease. Our research focuses on the role of these novel factors and their possible links with maternal periodontal disease.

Search Strategy

A thorough literature review was performed using electronic databases such as PubMed, Scopus, Google Scholar, Web of Science, and Embase. The search included keywords such as maternal periodontitis, maternal periodontal diseases, adverse pregnancy outcomes, preterm birth, preeclampsia, epigenetics, maternal stress, maternal vitamin D, genetics, innate immunity, and PRR. Studies considered included prospective/retrospective cohort studies, cross-sectional studies, case-control studies, review articles, and case series/reports published in English up to February 2024, which explored the potential connection between these factors and maternal periodontal diseases.

Epigenetics

Epigenetics deals with studying the alterations in the genetic expression with no changes in the DNA sequences [13]. The changes produced are heritable and can be transmitted from one generation to another. The epigenetic changes postulated to modulate the gene expression include (a) DNA methylation (b) histone modification, and (c) noncoding RNAs [14,15].

DNA methylation is a covalent bonding addition to the DNA, passed on from generation to generation. Histone modifications include additions to the amino and carboxy-terminal ends. The human genome encompasses sequences for both protein-coding RNAs (mRNAs) and noncoding RNAs (ncRNAs). As proteins serve as the principal functional output of genetic information, mRNAs have been extensively investigated, in contrast to their noncoding equivalents. Although most ncRNAs cannot be translated into protein, many play a role in maintaining vital biological functions by regulating transcription and posttranslational modifications [16].

Genomic imprinting, which in turn is controlled by DNA methylation, is the most extensively explored epigenetic phenomenon in the human placenta [17]. Humans inherit one allele maternally and one allele paternally. In some circumstances, one allele is “stamped” (or turned off) and does not show in the offspring, and that particular gene is said to be “imprinted” or silenced and is not expressed. Thus, genomic imprinting is a phenomenon when two alleles are morphologically equivalent but functionally diverse and can lead to the occurrence of parent-origin effects [18]. Epigenetic changes can occur early in development, such as LINE1 elements (long interspersed elements), and others can appear later at the H19/IGF2 locus. This has been revealed via DNA methylation analysis of genomic segments [19].

Any alternation in the maternal intrauterine environment (because of inflammation, environment, microbes, and others) may alter the epigenetic profile and gene expression and disrupt normal placental function. This defunct placental function could be implicated in pregnancy-related diseases such as adverse pregnancy outcomes and adverse fetal outcomes.

Stress

Stress is an established risk determinant for periodontal diseases. In the placental tissues, an increase in methylation at the HSD11B2 gene locus is implicated in increased levels of maternal stress in pregnant rats [20]. Stress has a direct neuro-immuno-endocrinological influence as it may trigger the activation of the hypothalamus and the autonomic nervous system resulting in an increased secretion of certain chemicals (e.g., cortisol). High cortisol levels reduce lymphocyte sensitivity to glucocorticoids by binding to glucocorticoid receptors. As steroid resistance develops, this leads to an increased release of pro-inflammatory cytokines. Moreover, maternal stress affects circulating levels of inflammatory markers by increasing pro-inflammatory cytokines IL-1β, IL-6, and TNF-α, while decreasing the anti-inflammatory cytokine IL-10. These inflammatory markers weaken the immune response, raising the risk of adverse pregnancy outcomes, such as preterm birth [21].

Chronic stress may also exhibit an indirect effect on the tooth-supporting tissues with an unfavorable influence on the occurrence, progression, and response to the treatment regimen of periodontal diseases. This may result from altered behavioral habits because of stress (smoking, poor oral hygiene, etc.). It is of paramount importance to delineate patients under chronic stress, thereby entailing multidisciplinary treatment strategies (e.g., physician, oral surgeon, and psychologist) to reduce the stress effects [22].

Vitamin D Status

Vitamin D is a pleiotropic hormone that primarily regulates serum calcium and phosphorus metabolism. Moreover, it exhibits antiproliferative, antiangiogenic, pro-differentiating, and pro-apoptotic activities. A distinctive nuclear hormone receptor, the vitamin D receptor (VDR), is chiefly accountable for its biological actions. Vitamin D regulates the cutaneous immune system homeostasis, differentiation, and proliferation of keratinocytes, and apoptotic mechanisms. Vitamin D exhibits anti-inflammatory actions and modulates the adaptive and innate immune response [23].

Vitamin D comes under the category of fat-soluble vitamin with its main natural source being cutaneous synthesis postexposure to solar rays. Preeclampsia is a condition wherein proteinuria and hypertension occur post 20 weeks of gestation and generally affect 4.6% of pregnancies worldwide [24]. Several studies including meta-analysis have been conducted to ascertain the relationship between vitamin D status and pregnancy outcomes. Recent literature has established that preeclampsia has a strong linkage with decreased 25(OH)D levels [24,25].

The latest guideline by the World Health Organization suggests recommending 25(OH)D supplementation for women with 25(OH)D deficiency during pregestational age, as it is preferred for preventing preeclampsia (PE). The US Institute of Medicine guidelines recommend a supplementation of 600 IU/day of vitamin D3 for pregnant women. However, the US Endocrine Society recommends maintaining serum concentrations of 25(OH)D above 30 ng/ml, with pregnant women requiring at least 600 IU/day supplementation. Notably, 1,500-2,000 IU/day of 25(OH)D may be necessary to maintain the serum 25(OH)D concentrations [26].

Evidence linking preterm birth and hypovitaminosis is less conclusive. A meta-analysis study showed that vitamin D levels of less than 50 nmol/L have a positive correlation with preterm birth [27]. Considering general obstetric patients, concentrations of vitamin D ≥40 ng/ml seem to pose a lesser risk of preterm birth by about 60% [28]. Keeping in view the cultural variations, the incidence of hypovitaminosis and preterm birth commonly occur among women belonging to the ethnic minority/non-white category [29]. However, conducted studies have shown inconsistent evidence between maternal 25(OH)D deficiency and birth weight [25,27,30].

Genetic Factors

Genes constitute the foundational basis of an individual. From the perspective of risk assessment of periodontal disease, genetic factors come under the category of background characteristics/risk determinants. These are those factors that are non-modifiable. Recent studies have firmly advocated the role of genetic factors as the potential risk factors for periodontitis. Several studies such as twin studies, family studies, population studies, and single nucleotide polymorphisms (SNP) may delineate the genetic origin of periodontitis. Gene polymorphisms such as interleukin-1 (IL-1) and tumor necrosis factor-α (TNF-α) may reveal a positive correlation with increased severity of periodontal tissue destruction [31].

With the implementation of sophisticated machinery, there has been tremendous development in genomic technology. Inflammation has a central role to play both in periodontal disease and in adverse pregnancy outcomes. It has been proved at various levels that inflammatory response is largely under genetic control. Hence, the search for an inflammatory genetic locus for both these disease entities is of paramount importance. Most of the information has been obtained from three types of genetic studies (a) candidate gene studies, (b) genome-wide association studies (GWAS), and (c) whole exome studies (WES) [30].

A recent GWAS with a sample size of 40,000 women of Caucasian descent revealed that maternal genetic loci such as early B-cell factor 1 (EBF1), eukaryotic elongation factor selenocysteine-tRNA specific (EEFSEC), and angiotensin II receptor type 2 (AGTR2) contribute to preterm birth [32]. EBF1 plays a role in B cell maturation and development, EEFSEC is associated with encoding a protein to produce selenoproteins, while the angiotensin II receptor is encoded by AGTR2 [32]. 

Role of Innate Immunity and Inflammation

Nucleotide-binding oligomerization domain-containing protein 1 (NOD1) is a receptor protein that recognizes foreign molecules. Nucleotide-binding oligomerization domain-containing protein 2 (NOD2) is another receptor protein that recognizes foreign molecules. Certain genes are coding for proteins, which are associated with lessening the effect of immune responses especially innate immune responses.

A recent whole exome sequencing (WES) recognized unusual genetic mutations, which code for proteins (negatively dampen the innate immune response) such as caspase recruitment domain-containing protein 6,8 encoded by CARD6, CARD8, nucleotide-binding oligomerization domain-containing protein (NOD2, toll-like receptors (TLR10) and anti-microbial peptide/proteins defensin beta 1 (DEFB1), mannose-binding lectin (MBL). This study was done from DNA extracts of preterm neonates in African American mothers after untimely membrane rupture. The findings of this study support the long-held theory that preterm births have an inflammatory component to their etiology.

The reasons could be because of pathogens or “alarmins” released during any stressful situation for the cell. Strauss et al. suggest that whole exome and whole genome sequencing appear to be the most lucrative perspective for recognizing functionally noteworthy biological variants concerning preterm birth and pathologic inflammation [30]. Whether these genetic mutations confer the same risk to other ethnic groups in other countries or continents and their association with adverse pregnancy outcomes and periodontal disease remains a subject of research. An overlap among periodontal disease, preterm birth, inflammatory bowel disease, and WES-recognized genes was detected, thereby advocating a genetic commonality among them [30].

Role of PRRs

The human immune system can identify pathogenic microbes through PRR, thereby initiating an immune reaction induced by several pro-inflammatory mediators. PRRs identify and react to conserved patterns developed in microorganisms and stress signals through damage-associated molecular patterns or pathogen-associated molecular patterns (DAMPS or PAMPS) [33].

The following two sets of PRR have been implicated in preterm birth:

(a) Inflammasomes in adverse pregnancy outcomes: The inflammasome is a protein entity that by the action of active caspase-1 promotes the conversion of the immature substrate of IL-1β into a mature form [34,35]. The role of this entity in preterm labor comes from the evidence of higher concentration of IL-1β, caspase-1, and IL-18 in women suffering from preterm labor with amniotic infection than those without it [36-38]. 

(b) Nucleotide oligomerization domain (Nod) proteins: A second set of PRR incriminated in the inflammatory pathological cascade of labor are the nucleotide oligomerization domain NOD1 and NOD2 proteins [39]. The membranes of the myometrium and chorioamnion express both NOD proteins during inflammatory pathological processes of preterm labor [40]. A peculiar quality of these intracellular molecules is to recognize peptidoglycan molecules and alarmins without the formation of inflammasomes [41,42]. Instead, these domain proteins cause direct stimulation of nuclear factor kappa B (NF-κB) cascade inducing the formation of pro-IL-1β and pro-IL-18 [43]. Some inflammasome components work synergistically with domain proteins to trigger an immune reaction in murine dendritic cells [44].

Paternal Status

Until now, all focus has been on the maternal and fetal factors when attempting to link periodontal disease with pregnancy outcomes. The role of paternal genetic status remains an enigma that needs to be further investigated. Moreover, what is the role of the periodontal status of the father, and is it in any way related to adverse pregnancy outcomes in the mother?

**Figure 2:**
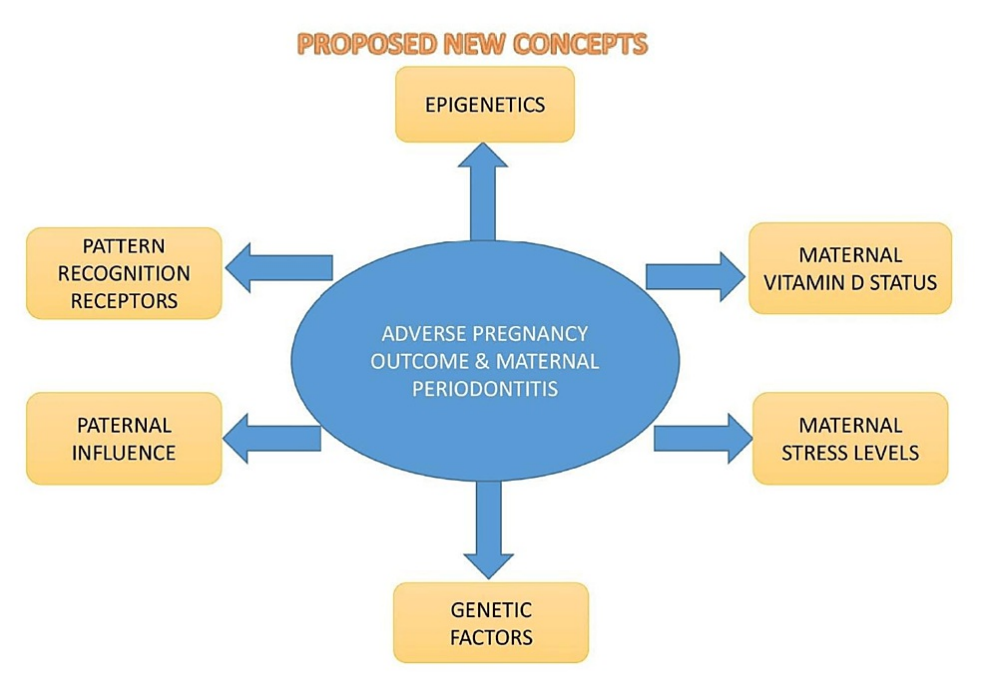
Proposed concept of possible links between maternal periodontitis and adverse pregnancy outcomes. Image credit: Dr. Sarah Mariam

Our review proposes newer concepts to explore the connection between maternal periodontitis and adverse pregnancy outcomes, as depicted in Figure 2.

## Conclusions

Periodontitis is a commonly occurring chronic inflammatory disease that can be easily preventable. However, early diagnosis and management are frequently ignored during pregnancy owing to the prevalent fallacies linked with the safety of treating pregnant women. Hence, emphasis should be placed on an early diagnosis and management of periodontal diseases, and routine oral health evaluation during prenatal care should be encouraged to combat complications.

Several studies have attempted to explore the possible relationship between maternal periodontal health and adverse pregnancy outcomes but with conflicting results. Ensuing endeavors should be undertaken to help find plausible mechanisms keeping in view the future research domains and new pathways. Upcoming research should focus on the plethora of genetic and inflammatory evidence in the context of predicting maternal and fetal pregnancy outcomes. Paternal factors such as genetic, innate, and inflammatory should be investigated in search of new evidence. The role of epigenetic factors has opened novel gateways that have in a way opened Pandora's box.
